# Image-Based Recurrence Patterns After Reirradiation in Prostate Cancer with Long-Term Follow-Up

**DOI:** 10.1016/j.adro.2025.101900

**Published:** 2025-09-16

**Authors:** Una Ryg, Wolfgang Lilleby, Line Brennhaug Nilsen, Taran Paulsen Hellebust, Therese Seierstad, Knut Håkon Hole

**Affiliations:** a Division of Radiology and Nuclear Medicine, Oslo University Hospital, Oslo, Norway; b Institute of Clinical Medicine, University of Oslo, Oslo, Norway; c Department of Oncology, Oslo University Hospital, Oslo, Norway; d Department of Medical Physics, Oslo University Hospital, Oslo, Norway; e Department of Physics, University of Oslo, Oslo, Norway

## Abstract

**Purpose:**

Local failure of prostate cancer after definitive radiation therapy is associated with poor prognosis. Studies on reirradiation have primarily focused on toxicity and oncologic outcome and only partially reported recurrence patterns. Investigating the recurrence pattern may help guide future therapy decisions.

**Methods and Materials:**

Thirty-three men with local recurrence of prostate cancer after primary definitive radiation therapy were enrolled between 2012 and 2018 (median age 69.8 years [IQR: 6.8], median prostate-specific antigen 4.1 ng/mL [IQR: 3.8]). Twenty-three patients received reirradiation with focal high dose-rate brachytherapy, and 10 received stereotactic body radiation therapy to the prostate with (8/10) or without (2/10) a simultaneous integrated boost to the recurrent tumor. The sites of recurrences were examined with multiparametric magnetic resonance imaging and compared with the dose distribution maps.

**Results:**

During the median 99 months (IQR: 56) follow-up, 25 patients had biochemical rerecurrence. Twenty had adequate imaging. Five patients had rerecurrences solely inside the high-dose region, and 7 had both inside and outside the high-dose region. Two patients with a prostatic recurrence received whole-gland stereotactic body radiation therapy without a boost to the tumor. Four had a combination of rerecurrence within the prostate as well as regional lymph node metastases. One patient had a prostatic rerecurrence and a single bone metastasis. One patient had prostatic rerecurrence, lymph node metastases, and bone metastases. No patients had only regional or distant metastases.

**Conclusions:**

After reirradiation of prostate cancer, the tumor frequently recurred within the prostate, both inside and outside the high-dose region. About 1 in 3 patients also had regional or distant metastatic disease at rerecurrence.

## Introduction

Local failure after definitive radiation therapy is associated with a poor prognosis,^1^^,^^2^ and is one of the most common recurrence sites after definitive radiation therapy.^3^^,^^4^ Within the prostate, the local recurrences usually occur in the same site as the primary lesion,^5^, ^6^, ^7^, ^8^ indicating that the initial radiation dose was too low to ablate all cancer cells.

There is no current consensus on how to best treat locally recurrent prostate cancer after radiation therapy.^9^^,^^10^ Potential curative strategies include radical prostatectomy, high-intensity focused ultrasound, cryoablation, brachytherapy (BT), and stereotactic body radiation therapy (SBRT).^11^, ^12^, ^13^ These strategies have comparable relapse-free survival rates, but reirradiation appears to have a lower rate of severe genitourinary and gastrointestinal toxicity.^9^^,^^10^

Most patients with radiorecurrent prostate cancer receive androgen deprivation therapy (ADT),^14^, ^15^, ^16^ which has considerable side effects^17^^,^^18^ and does not cure the patient. Therefore, local reirradiation may be underused.^16^

There are limited recommendations regarding modality, dose, target volume delineation, and technique for reirradiation of locally radiorecurrent prostate cancer.^19^, ^20^, ^21^ Studies on reirradiation have primarily focused on toxicity and oncologic outcome and have only partially reported the pattern of failure. Understanding the failure pattern may help guide future therapy decisions. The aim of this study was to examine the long-term pattern of failure after reirradiation.

## Methods and Materials

### Study population

This study is a follow-up of a prospective phase 2 trial that examined the long-term toxicity and clinical outcome of patients treated with salvage high-dose-rate BT (HDR-BT) or SBRT for locally radiorecurrent prostate cancer.^22^

The inclusion criteria for this study were as follows: biochemical failure as described in the Phoenix definition, with a prostate-specific antigen (PSA) elevation of nadir + 2 ng/mL,^23^ PSA below 10 ng/mL, a PSA doubling time above 6 months, more than 2 years of recurrence-free interval after primary radiation therapy, Eastern Cooperative Oncology Group (ECOG) performance status 0-1^24^ with a life expectancy of more than 5 years, and no evidence of metastasis at imaging or in bone marrow aspiration.

**Figure 1:**
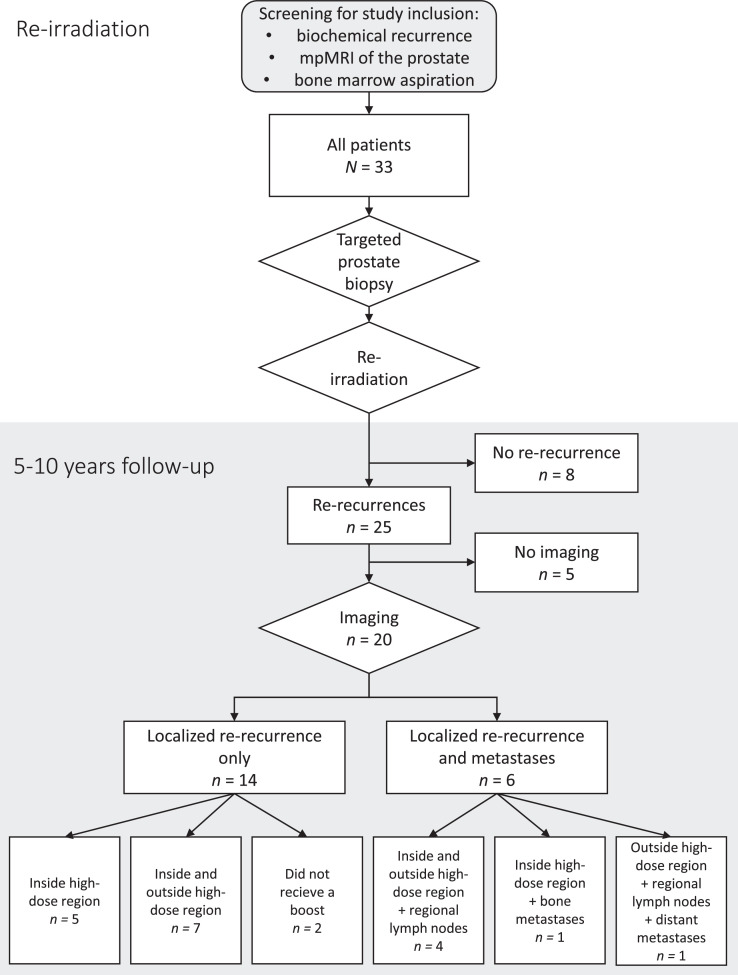
Study design. Flow diagram of the study.

Thirty-three men with local recurrence of prostate cancer after primary definitive radiation therapy were enrolled consecutively between April 2012 and December 2018 (Fig. 1). The median age at inclusion was 69.8 years, with an IQR of 6.8 years. The median PSA was 4.1 ng/mL (IQR: 3.8 ng/mL). The median time between the initial radiation therapy and the diagnosis of the local recurrence was 73 months (IQR: 46 months). Multiparametric magnetic resonance imaging (MRI) of the pelvis and lower lumbar spine was acquired for all patients. Twenty-four patients were also examined with whole-body ^18^F-fluciclovine positron emission tomography (PET)/computed tomography (CT); 2 had prostate-specific membrane antigen (PSMA) PET/CT; and 3 had whole-spine MRI. Four patients had no additional imaging. Eleven patients were intermediate and 22 were high risk at primary treatment according to the D'Amico risk classification.^25^ Patient characteristics at baseline and follow-up are shown in Tables 1 and 2. Seven of the men with high-risk disease received radiation therapy to both the pelvis and prostate concomitantly at primary diagnosis as part of a different study.^26^ Three of them had regional lymph node metastases at the initial diagnosis but had no evidence of metastases at reirradiation. At primary treatment, 25 received ADT, 22 with luteinizing hormone-releasing hormone agonist, and 2 with nonsteroidal antiandrogen monotherapy (patients 4 and 19) (Table 1). Prostate biopsy at inclusion verified local recurrence in all except one of the patients. This patient had evidence of local recurrence on MRI in the left lobe, while targeted biopsy cores unfortunately were sampled from the right lobe.^22^ Five patients (15.2%) received either ongoing or concomitant luteinizing hormone-releasing hormone agonist at reirradiation. Rerecurrences were defined as biochemical recurrences using the Phoenix criteria with nadir PSA after reirradiation.^23^ After rerecurrence, 22 of 25 patients received ADT (Table 2).

**Table 1:** Overview of patient characteristics and treatment at primary diagnosis

Patient	iGS	T	N	M	iPSA (ng/mL)	D'Amico risk group	RT (Gy)	ADT (mo)
1	4 + 3	T2c	0	0	14.7	High	74 Gy	0
2	3 + 4	T2b	0	0	8	Intermediate	74 Gy	0
3	3 + 4	T2c	0	0	10	High	74 Gy	12
4	3 + 4	T1c	0	0	22	High	74 Gy	3
5	4 + 5	T3b	0	0	59	High	74 Gy	36
6	3 + 3	T1c	0	0	42	High	74 Gy	24
7	4 + 3	T3b	0	0	66	High	74 Gy*	24
8	2 + 3	T1c	0	0	11.3	Intermediate	74 Gy	0
9	4 + 5	T3b	0	0	58	High	74 Gy*	24
10	3 + 3	T2b	0	0	18	Intermediate	74 Gy	0
11	5 + 4	T3b	1	0	39	High	74 Gy*	24
12	4 + 3	T3a	0	0	45	High	74 Gy	30
13	3 + 4	T2b	0	0	13	Intermediate	74 Gy	0
14	3 + 3	T1c	0	0	12	Intermediate	74 Gy	6
15	3 + 4	T2a	0	0	4.5	Intermediate	74 Gy	6
16	3 + 4	T3a	0	0	28	High	74 Gy	30
17	3 + 4	T2c	0	0	30	High	70 Gy	0
18	4 + 4	T3b	1	0	17	High	74 Gy*	36
19	3 + 4	T2a	X	0	20	Intermediate	70 Gy	12
20	3 + 3	T1c	X	0	10	Intermediate	74 Gy	0
21	3 + 4	T3a	0	0	5.4	High	74 Gy	24
22	3 + 3	T2b	0	0	15.5	Intermediate	74 Gy	0
23	4 + 4	T3a	0	0	9.4	High	74 Gy	24
24	4 + 4	T3a	0	0	17	High	74 Gy	24
25	3 + 4	T2b	X	0	8	Intermediate	78 Gy	6
26	3 + 5	T3a	0	0	70	High	74 Gy	24
27	3 + 4	T3a	X	0	6.2	High	70 Gy	6
28	3 + 4	T2c	0	0	44	High	74 Gy	24
29	4 + 4	T3b	1	0	7.6	High	74 Gy*	48
30	3 + 4	T3b	0	0	29	High	74 Gy*	24
31	4 + 3	T3a	0	0	10	High	74 Gy	24
32	4 + 3	T2c	0	0	8	Intermediate	74 Gy	12
33	4 + 4	T3b	0	0	37	High	74 Gy*	24

*Abbreviations:* ADT = androgen deprivation therapy; iGS = initial Gleason score; iPSA = initial PSA; PSA = prostate-specific antigen; RT = radiation therapy; X = NX-staging at diagnosis.

⁎ Received concomitant radiation therapy to pelvic lymph nodes.

**Table 2:** Overview of patient characteristics and treatment at reirradiation

	At reirradiation								After rerecurrence
Patient	Age (years)	PSA (ng/mL)	ADT (mo)	Modality and fractionation regime (Gy)		Nominal total dose (Gy)	GTV EQD2 α/β=3 Gy (Gy)	nPSA (ng/mL)	ADT
1	70.7	6.4	0	HDR-BT	10 Gy x 3	30 Gy	78 Gy	1.0	Yes
2	66.4	4.2	0	HDR-BT	10 Gy x 3	30 Gy	78 Gy	0.3	N/A
3	68.2	2.8	0	HDR-BT	10 Gy x 3	30 Gy	78 Gy	0.2	N/A
4	65.2	3	0	HDR-BT	10 Gy x 3	30 Gy	78 Gy	1.9	No
5	58.3	2.3	0	HDR-BT	10 Gy x 3	30 Gy	78 Gy	2.9	Yes
6	65.7	4	0	HDR-BT	10 Gy x 3	30 Gy	78 Gy	0.5	N/A
7	68.3	3.8	0	HDR-BT	10 Gy x 3	30 Gy	78 Gy	1.8	Yes
8	66.7	1.9	0	HDR-BT	10 Gy x 3	30 Gy	78 Gy	0.6	Yes
9	66.1	3.3	0	HDR-BT	10 Gy x 3	30 Gy	78 Gy	0.7	Yes
10	64.7	4.5	0	HDR-BT	10 Gy x 3	30 Gy	78 Gy	0.7	Yes
11	70.4	7.9	60	HDR-BT	10 Gy x 3	30 Gy	78 Gy	0.2	Yes
12	67.8	4.5	0	HDR-BT	10 Gy x 3	30 Gy	78 Gy	3.9	Yes
13	64.0	6.5	0	HDR-BT	10 Gy x 3	30 Gy	78 Gy	0.6	No
14	69.8	7.2	0	HDR-BT	10 Gy x 3	30 Gy	78 Gy	0.5	No
15	68.3	1.6	0	HDR-BT	10 Gy x 3	30 Gy	78 Gy	0.2	Yes
16	63.6	8.5	0	HDR-BT	10 Gy x 3	30 Gy	78 Gy	1.9	Yes
17	72.3	9.6	0	HDR-BT	10 Gy x 3	30 Gy	78 Gy	1.5	Yes
18	74.9	6.4	0	HDR-BT	10 Gy x 3	30 Gy	78 Gy	7.5	Yes
19	69.9	4.2	0	HDR-BT	10 Gy x 3	30 Gy	78 Gy	1.2	Yes
20	72.9	6.5	0	HDR-BT	10 Gy x 3	30 Gy	78 Gy	0.7	Yes
21	72.4	4.7	0	HDR-BT	10 Gy x 3	30 Gy	78 Gy	1.5	Yes
22	74.3	4	3	HDR-BT	10 Gy x 3	30 Gy	78 Gy	0.0	N/A
23	77.0	4.1	3	HDR-BT	10 Gy x 3	30 Gy	78 Gy	0.1	Yes
24	68.6	5	0	SBRT	7(5) Gy x 5	35(25) Gy	70 Gy	0.7	Yes
25	72.5	3.5	0	SBRT	7(5) Gy x 5	35(25) Gy	70 Gy	0.4	Yes
26	73.7	7.8	0	SBRT	7(5) Gy x 5	35(25) Gy	70 Gy	0.1	N/A
27	73.7	1.9	0	SBRT	7(5) Gy x 5	35(25) Gy	70 Gy	0.1	N/A
28	78.1	2.5	0	SBRT	7(5) Gy x 5	35(25) Gy	70 Gy	0.2	N/A
29	67.1	0.83	26	SBRT	5 Gy x 6	30 Gy	48 Gy	0.7	Yes
30	75.1	0.31	6	SBRT	7(5) Gy x 5	35(25) Gy	70 Gy	0.0	N/A
31	67.4	0.6	0	SBRT	7(5) Gy x 5	35(25) Gy	70 Gy	0.6	Yes
32	71.6	3.9	0	SBRT	5 Gy x 5	25 Gy	40 Gy	0.8	Yes
33	75.5	4.6	0	SBRT	7(5) Gy x 5	35(25) Gy	70 Gy	2.7	Yes

*Abbreviations:* ADT = androgen deprivation therapy; BT = brachytherapy; EQD2 = equivalent dose in 2 Gy fractions; GTV = gross tumor volume; HDR = high dose rate; N/A = not applicable (no rerecurrence); nPSA = nadir prostate-specific antigen; PSA = prostate-specific antigen; SBRT = stereotactic body radiation therapy.

The regional ethics board approved this study (REK 284446) and waived the need for a separate consent based on the previous consent from the prospective study (REK 2011/954).

### Salvage radiation therapy

The first 23 patients received salvage HDR-BT, and the following 10 received SBRT. The change from HDR-BT to SBRT was physician driven. The HDR-BT procedure is described in detail in references^22^ and^27^, and the SBRT procedure is described in detail in reference^22^.

The gross tumor volume (GTV) was defined by the recurrent tumor visible on the MRI. For the patients receiving focal HDR-BT, the planning aim to the GTV was 30 Gy, delivered in 3 consecutive fractions of 10 Gy 2 weeks apart. The procedure was ultrasound guided, with inverse plan optimization. Organs at risk were the rectal wall and the urethra. SBRT was delivered on a linear accelerator with volumetric arc therapy to the whole prostate gland, defined as the clinical target volume (CTV), without (n = 2) or with (n = 8) a simultaneous integrated boost to the GTV. An isotropic margin of 3 mm was added to the CTV/GTV (if present) to provide the planning target volume(s). For the patients receiving simultaneous integrated boost, the total dose to the GTV/CTV was 35 Gy/25 Gy, delivered in 5 fractions. Two SBRT patients received either 6 fractions (patient 29) or 5 fractions (patient 32) of 5 Gy to the CTV. All SBRT fractions were given every other day, using fiducial markers for image guidance. The organs at risk were the urethra, bladder, rectum, anal canal, and femoral heads. The modality, fractionation schedule, and 2 Gy equivalent dose for each patient at the initial recurrence are given in Table 1. No patients had spacer gel placement. It is important to emphasize that HDR-BT delivers dose only to the GTV, while SBRT delivers dose to the whole prostate with a boost to the GTV.

### Image acquisition and interpretation

Multiparametric MRI prior to study inclusion was performed at different hospitals, but all examinations included morphologic T2-weighted, diffusion-weighted, and dynamic contrast-enhanced sequences of the prostate. Image quality met or exceeded the minimum standard specified by the Prostate Imaging for Recurrence Reporting guidelines for assessing radiorecurrence.^28^

**Figure 2:**
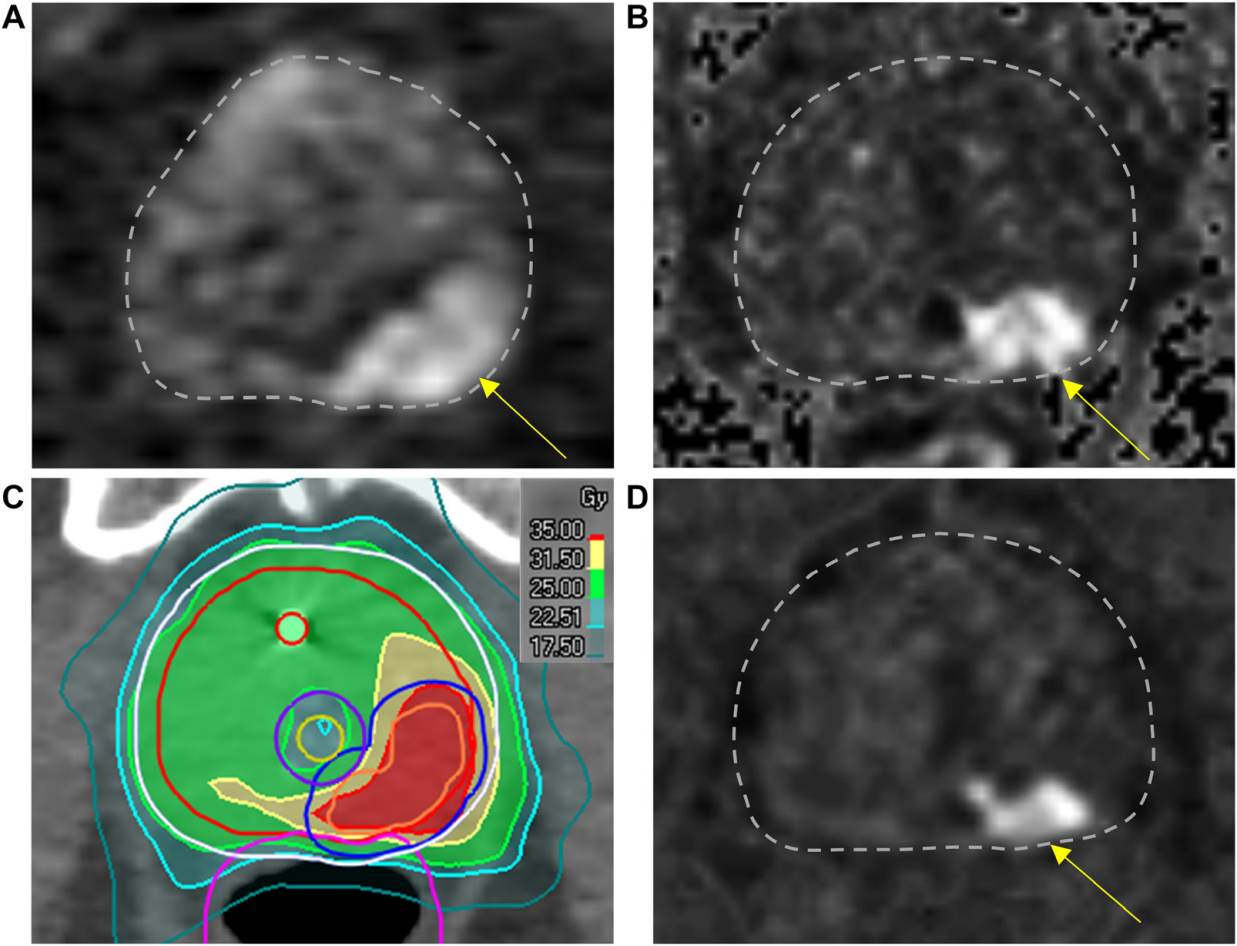
Site of local rerecurrence. Transversal diffusion-weighted images of the prostate at primary diagnosis (A), first recurrence (B) and rerecurrence (D) with the dose distribution from the corresponding image slice at reirradiation (C). Arrows point at tumor in the posterior left region of the prostate.

The local hospitals initiated imaging at rerecurrence. The exams included MRI of the pelvis and lower lumbar spine for 19 of the patients, of which 11 had additional imaging: MRI whole spine (n = 2), MRI torso (n = 7), ^18^F-fluciclovine PET/CT (n = 1), scintigraphy (n = 1). One patient had whole-body PSMA PET/CT (patient 33). A radiologist with 5 years of experience in prostate imaging (U.R) retrospectively compared these images (Fig. 2D) with those from the initial diagnosis (Fig. 2A), the first recurrence (Fig. 2B), and the reirradiation dose distributions (Fig. 2C) from the HDR-BT or the SBRT treatment plans. The images and the dose plans were coregistered and interpreted visually. The aim was to determine whether the rerecurrence was localized inside or outside the high-dose region, which we defined as the SBRT boost volume or the HDR-BT GTV (100% isodose line). We also recorded whether the local rerecurrences appeared in the same region as the first tumor recurrence. Images of the comparisons of each patient are provided in Supplementary material. The MRI interpretation of recurrences corresponds to the assessment categories 4 and 5 in the recently published Prostate Imaging for Recurrence Reporting system for MRI assessment of local prostate cancer recurrence after radiation therapy.^28^ In the definition of recurrence patterns, we included regional lymph nodes and distant metastases.

### Statistical analyses

The data are presented with descriptive statistics, carried out with StataCorp. 2023 (*Stata Statistical Software: Release 18*, College Station, TX: StataCorp LLC, RRID: SCR_012763). Biochemical recurrence-free survival, overall survival, and prostate cancer-specific survival were calculated with Kaplan-Meier analyses from the date of the first fraction of reirradiation. Death because of prostate cancer was defined as death in a patient with hormone-refractory metastatic prostate cancer, no other obvious cause of death, and evidence of rising PSA at the last follow-up. Patients were, as a rule, censored at the date of biochemical rerecurrence, at the date of death, or at the last follow-up. One patient was diagnosed with metastatic malignant melanoma in 2017 and had no prostate cancer-specific follow-ups between 2017 and 2022, when PSA had reached 12 ng/mL. In the survival analysis, he is censored at the date of the last prostate cancer-specific follow-up (patient 13). One patient with rising PSA had rerecurrence on imaging before he reached the Phoenix definition. For this patient, rerecurrence date is set to the MRI examination (patient 24).

Figures were created in StataCorp. 2023, Microsoft Office PowerPoint 2016 (RRID: SCR_023631), and Microsoft Office Excel 2016 (RRID: SCR_016137).

## Results

**Figure 3:**
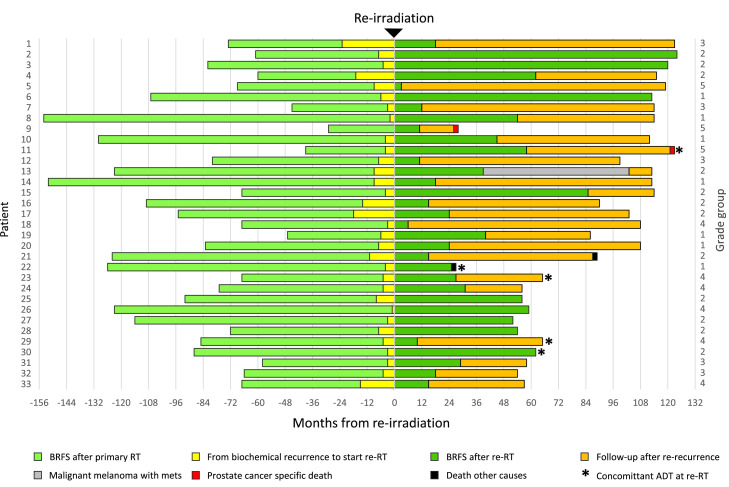
Patient event history. Swimmer plot of patient event history. *Abbreviations*: ADT = androgen deprivation therapy; BRFS = biochemical recurrence-free survival; RT = radiation therapy; with mets = with metastases.

Median follow-up was 99 months (IQR: 56 months). Median follow-up for recurrence-free patients was 60.5 months (IQR: 63.5 months). During the follow-up period, 25 patients had biochemical rerecurrence. Of these, 20 had adequate imaging of the rerecurrence site (Fig. 1). A swimmer plot of the event history, follow-up time, and biochemical recurrences for all patients is provided in Fig. 3.

Of 25 patients, 19 were examined with MRI at rerecurrence (Fig. 1). One patient was not examined with MRI at rerecurrence, but PSMA PET/CT showed marked uptake in the anterior part of the prostate, indicative of tumor rerecurrence (Supplementary material, patient 33). Figure 1 summarizes the pattern of rerecurrences based on whether they were inside or outside the reirradiation high-dose region, in regional lymph nodes, as distant metastases, or a combination. Five patients had rerecurrences solely inside the high-dose region, and 7 had both inside and outside the high-dose region. Two patients with a prostatic recurrence received whole-gland SBRT without a boost to the tumor (patients 29 and 32). Four had a combination of rerecurrence within the prostate as well as regional lymph node metastases. One patient had a prostatic rerecurrence and a single bone metastasis. One patient had prostatic rerecurrence, lymph node metastases, and bone metastases. No patients had only regional or distant metastases. A numeric summary of the failure sites is listed in Table 3.

**Table 3:** Sites with evidence of cancer at rerecurrence

Patient	Inside high-dose region	Outside high-dose region	Regional lymph node metastases	Bone metastases
1	X			
4	X			
5	X	X	X	
7	X	X	†	
8	X			
9	X	X*	†	
10		*No prostate imaging at rerecurrence*		
11		*Inadequate imaging at rerecurrence*†		
12	X	X‡	X	
13	*No prostate imaging at rerecurrence*			
14	X	X		
15	X	X		
16	X			X
17	*No prostate imaging at rerecurrence*			
18	X		†	
19	X			
20	X	X		
21	X	X*		
23	X	X		
24	X	X	X	
25	*No prostate imaging at rerecurrence*			
29	X§		†	
31	X	X	X	
32		X§		
33		X*	X†	X

⁎ Boost field did not cover the extent of the first recurrence.

† Received concomitant pelvic irradiation at primary diagnosis.

‡ In a region of periurethral dose sparing.

§ Did not receive boost to first recurrence.

All 20 patients with imaging at rerecurrence had a rerecurrence component in the same site as the first recurrence. For 3 patients, the reirradiation high-dose region did not fully cover the extent of the first recurrence (patients 9, 21, and 33). These 3 patients had persistent tumor here at follow-up. Similarly, we also detected a rerecurrence in a region of peri-urethral dose sparing in another patient (patient 12). Presentations of imaging and dose plans for individual patients are provided in Supplementary material.

Biochemical recurrence-free survival rates at 5 and 10 years were 29.1% (95% CI, 14.0%-46.1%) and 18.2% (95% CI, 5.7%-36.2%) (Supplementary material). Four patients died, 2 from prostate cancer and 2 from other causes, of whom 1 was recurrence-free at the time of death (patient 22). Overall survival rates at 5 and 10 years were 93.9% (95% CI, 77.9%-98.5%) and 89.2% (95% CI, 69.4%-96.5%), while prostate cancer-specific survival rates at both 5 and 10 years were 96.9% (95% CI, 79.8%-99.6%) (Supplementary material). Of note, toxicity, dose to organs at risk and oncologic outcome at a median follow-up of 81 months were reported previously.^22^

## Discussion

In this study, we investigated the rerecurrence patterns in patients who had undergone focal reirradiation for locally radiorecurrent prostate cancer. Of 33, 25 patients (76%) experienced a second biochemical relapse. Of these, 20 had imaging of the rerecurrence site. All 20 had rerecurrence within the prostate. Six also had lymph node and/or bone metastases (Fig. 1).

Several studies on reirradiation have to some extent included information on whether the local recurrences occurred inside or outside the reirradiation field,^29^, ^30^, ^31^, ^32^, ^33^, ^34^, ^35^, ^36^ but to the best of our knowledge, only Rasing et al^37^ have examined the localization of intraprostatic recurrences after reirradiation in detail.

### Prostatic rerecurrence within the high-dose region

It is well documented that local recurrences of prostate cancer after radiation therapy usually occur at the same site as the primary tumor,^5^, ^6^, ^7^, ^8^ but less is known about the recurrence pattern after reirradiation. Of the 18 patients who received a high dose to the tumor, all had a rerecurrence component inside this high-dose region. Rasing et al^37^ analyzed the pattern of recurrence in 83 patients with a second relapse after focal salvage single-fraction HDR-BT and found a rerecurrence component inside the radiation field in 55 of 67 (82%) prostatic relapses. Greco et al reported the pattern of rerecurrence in 15 patients with a second relapse after salvage stereotactic ablative reirradiation of the prostate with a boost to the recurrent intraprostatic lesion. In their study, 7 of 8 (87.5%) prostatic relapses had a rerecurrence component at the same site as the initially recurrent intraprostatic lesion.^35^ Other studies looking at the site of local recurrence after reirradiation of prostate cancer have also found that the tumor commonly recurs within the radiation field.^29^, ^30^, ^31^^,^^33^^,^^36^

### Prostatic rerecurrence outside the high-dose region

In our study, 12 patients of 18 (67%) had a rerecurrence component within the prostate but outside the high-dose region (Fig. 1). Our findings are slightly higher than those of Rasing et al, who found that 34 of 67 (51%) prostatic rerecurrences to some extent had a tumor component outside the reirradiation field.^37^ Similarly, our findings are also slightly higher than those reported by Murgic et al, who investigated focal salvage HDR-BT of radiorecurrent prostate cancer in 15 patients and found that 3 of 6 (50%) rerecurrences were outside the HDR-BT field.^34^ Our results are in line with those reported by Pasquier et al, who found that of 10 prostatic rerecurrences, 6 (60%) were outside the reirradiation field.^30^ Overall, it appears that prostatic rerecurrences are relatively common also outside the high-dose region. The differences in reported occurrence rates may be because of variations in target volumes and the degree of dose escalation in the different studies. Recommendations for target volume at reirradiation are limited because few studies have directly compared whole-gland versus focal therapy, as highlighted by Andring et al.^38^

### Lymph node and bone metastases

Of the 20 patients with imaging at biochemical rerecurrence, 6 (30%) had developed lymph node or bone metastases. Rasing et al reported that after 5 years, 38 of 83 (46%) had regional or distant metastases. Of note, they stated that about half of the patients were treated off protocol, with relatively more high-risk tumor characteristics.^37^ Greco et al had a high rate of extraprostatic rerecurrences in their material; 11 of 15 (73%) patients had extraprostatic disease at the second biochemical relapse, but almost a quarter of the patients were already oligometastatic at inclusion.^35^ Miszczyk et al found a larger proportion of distant metastases than we did. In their material, 17 of 20 (85%) patients with rerecurrence developed distant metastases during follow-up. However, they also included oligometastatic as well as patients with castrate-resistant prostate cancer in their population. Castrate-resistant prostate cancer is a predictor of early metastases and poorer outcomes.^32^ Seven of our high-risk patients received concomitant radiation therapy to the lymph nodes at primary treatment (Table 1), likely influencing the incidence of lymph node metastases at rerecurrence. We may have missed some distant metastases as 8 patients had no additional imaging outside the pelvis and lower lumbar region, but our results are comparable with other studies that have reported rerecurrence patterns and indicate that about 30% to 40% have metastatic disease at rerecurrence.^29^^,^^33^^,^^36^^,^^39^, ^40^, ^41^, ^42^

### Clinical perspectives

Rerecurrence within the high-dose region indicates that neither the primary nor the reirradiation dose was sufficient for eradicating the tumor. An even higher dose could be required to kill the tumor but needs to be balanced against the toxicity to neighboring organs. Frequent rerecurrences within the prostate, but outside the high-dose radiation region, suggest that targeting only the visible tumor is insufficient. However, the aim of focal treatment at recurrence is not only to eradicate the tumor but to postpone ADT treatment. The rationale behind targeting the dominant lesion in the prostate stems from the concept that it contains the most aggressive cancer and is responsible for driving eventual metastatic disease.^43^^,^^44^ Even in a population of high-risk patients with a high relapse rate in a 5- to 10-year perspective, reirradiation likely delays the onset of ADT. Some of the patients developed metastatic disease, emphasizing the importance of patient selection.^45^ Markers for better patient selection are warranted,^21^ but heterogeneous tumor biology, different treatment methods, and small cohorts make it difficult to identify predictive markers.

### Limitations and strengths

This study has several limitations. The population is relatively small, with only 33 patients, and without controls. Also, the dose outside the high-dose region depended on the modality used for reirradiation. Biopsy was performed as a rule at the first recurrence, but not on rerecurrence. We can therefore neither confirm the persistence of viable tumor in the areas interpreted as positive nor exclude tumor outside of these areas.^46^ To mitigate this, all MRI findings, with corresponding dose distribution curves, are available as Supplementary material online. The strengths of this study include a long follow-up time, extensive imaging, and standardized bone marrow aspirations to exclude disseminated tumor cells in the bone marrow.

## Conclusions

After reirradiation in prostate cancer, the tumor frequently recurred within the prostatic gland, both inside and outside the high-dose region. Of the 20 patients who underwent imaging at rerecurrence, 6 (30%) had regional or distant metastatic disease in addition to local recurrence. Of 33, 7 (21%) of the reirradiated patients remained long-term recurrence free.

## Disclosures

The authors declare that they have no known competing financial interests or personal relationships that could have appeared to influence the work reported in this paper.

## Appendix Supplementary materials

Supplementary_sites_of_local_rerecurrence Image, application 1

Survival plots_anon Image, application 2

## Footnotes

Supplementary material associated with this article can be found in the online version at doi:10.1016/j.adro.2025.101900.
