# Supplementary material for: Image-Based Recurrence Patterns After Reirradiation in Prostate Cancer with Long-Term Follow-Up
Source: Adv Radiat Oncol. 2025 Sep 16;10(12):101900. doi: 10.1016/j.adro.2025.101900 (PMC12596965; doi:10.1016/j.adro.2025.101900)
Supplement: Supplementary_sites_of_local_rerecurrence [file mmc1.pdf]

Sites of local re-recurrence

Transversal DWI images with high b value show the sites of the primary intraprostatic lesion, the first recurrence, and the re-recurrence, along with the dose distribution from the corresponding image slice at re-irradiation. T2W images and/or dynamic contrast-enhanced (DCE) images are used when DWI was not available or had very sparse findings.

The prostate is delineated with a dashed line on the MR images. Solid arrows point at the primary intraprostatic lesion and recurrent lesions.

For the HDR-BT dose distributions, both the whole prostate and the recurrent tumor volume (GTV) have been delineated in blue, red, or white bold lines. The 120%, 100%, and 50% isodose lines are shown in thin turquoise, red, and green lines, respectively.

For the SBRT dose distributions, the prostate CTV is shown in red, the PTV to the prostate CTV is shown in white, the tumor GTV is shown in orange, and the PTV to the tumor GTV is shown in dark blue.

Abbreviations:

- DWI: Diffusion weighted images
- Re - RT: re-irradiation therapy
- HDR-BT: high dose-rate brachytherapy
- SBRT: Stereotactic body radiation therapy

Site of re-recurrence: patient 1

Primary lesion | First recurrence | Dose distribution at Re - RT | Re-recurrence

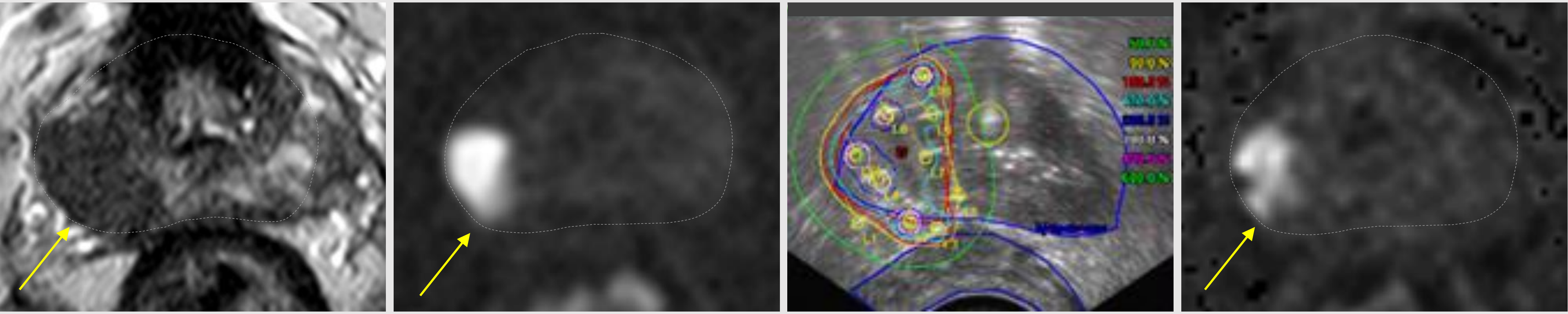

Transversal T2W of the primary lesion, DWI of the first recurrence, corresponding HDR-BT dose distribution, and DWI of the re-recurrence.

Re-recurrence in the same site as the primary lesion and the first recurrence, within the re-irradiation high-dose region.

Site of re-recurrence: patient 2

Primary lesion	First recurrence	Dose distribution at Re - RT	No re-recurrence
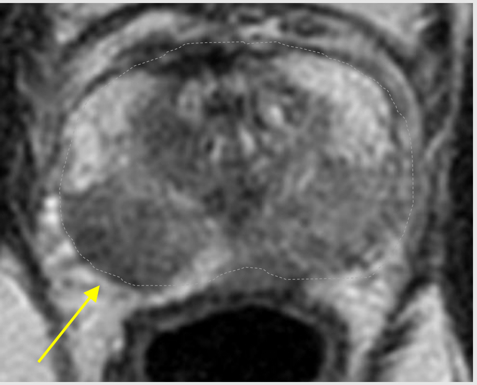	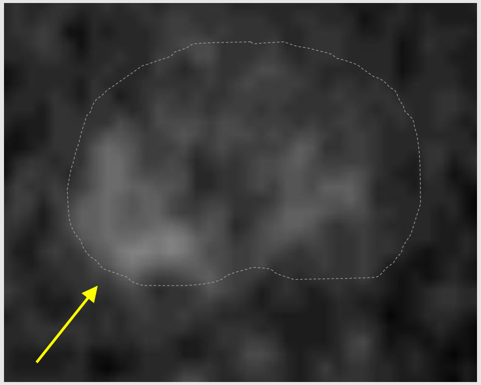	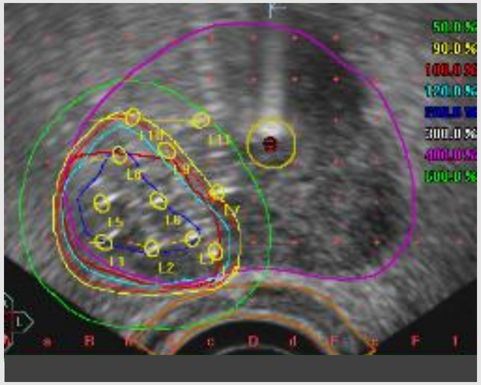	No re-recurrence

Transversal T2W of the primary lesion, DWI of the first recurrence, and corresponding HDR-BT dose distribution.

No re-recurrence.

Site of re-recurrence: patient 3

Primary lesion	First recurrence	Dose distribution at Re - RT	Re-recurrence
No prostate imaging	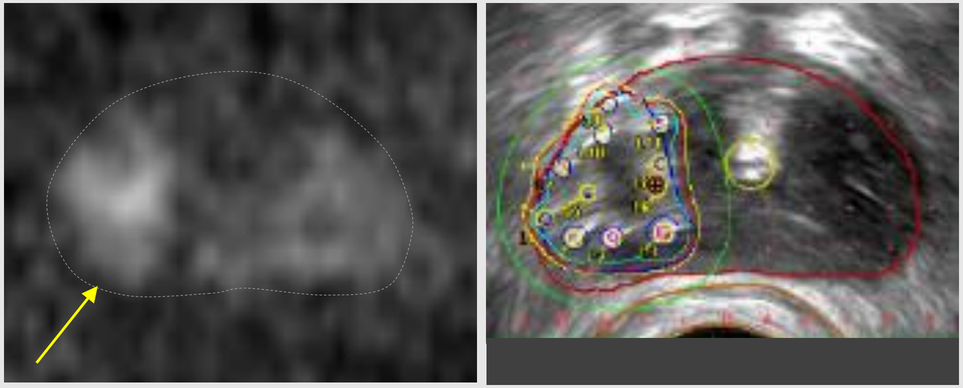		No re-recurrence

Transversal DWI and corresponding HDR-BT dose distribution.

No re-recurrence.

Site of re-recurrence: patient 4

Primary lesion	First recurrence	Dose distribution at Re - RT	Re-recurrence
No prostate imaging			

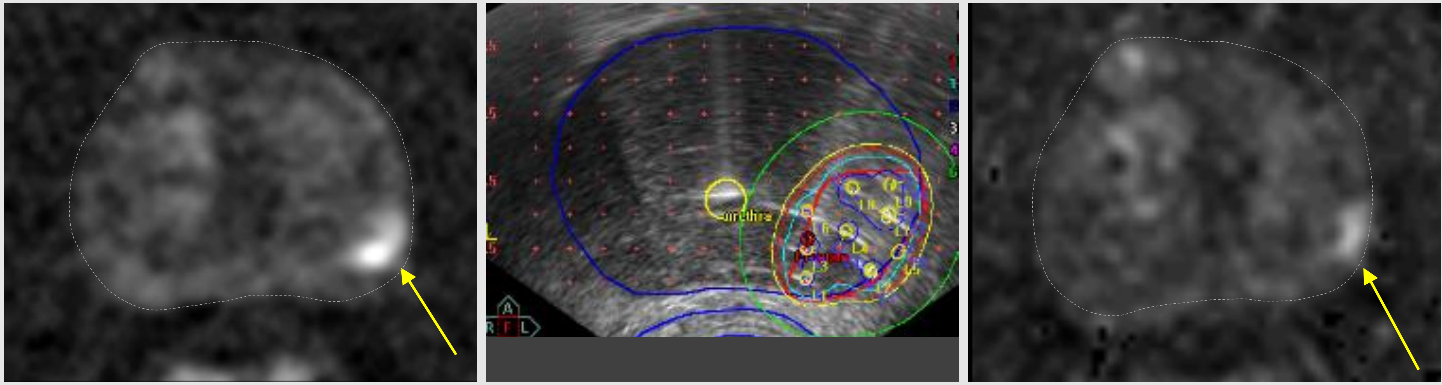

Transversal DWI and corresponding HDR-BT dose distribution.

Re-recurrence in the same site as the first recurrence.

Site of re-recurrence: patient 5

Primary lesion | First recurrence | Dose distribution at Re - RT | Re-recurrence

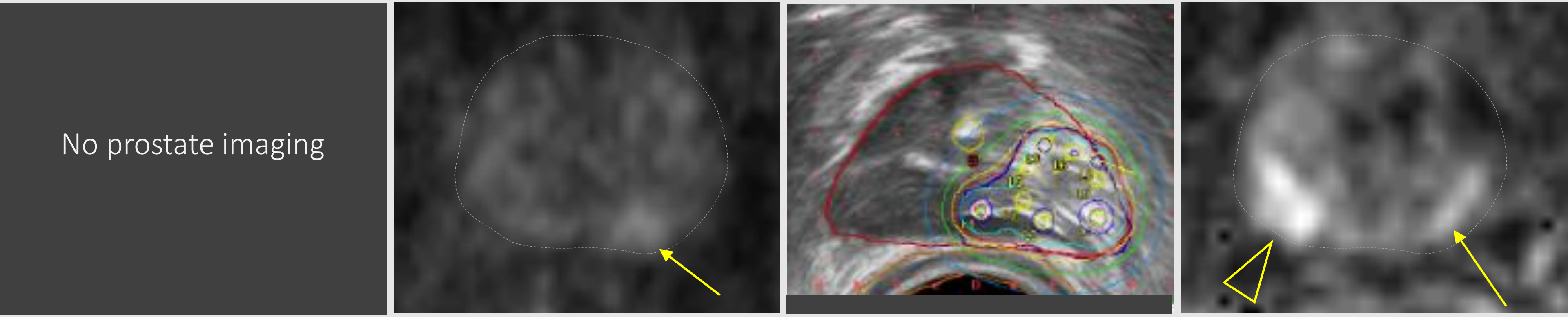

Transversal DWI at first and re-recurrence with corresponding HDR-BT dose distribution.

Re-recurrence in the same site as the first recurrence (arrows), and in a new site outside the re-irradiation high-dose region (arrowhead)

Site of re-recurrence: patient 6

Primary lesion	First recurrence	Dose distribution at Re - RT	Re-recurrence
No prostate imaging	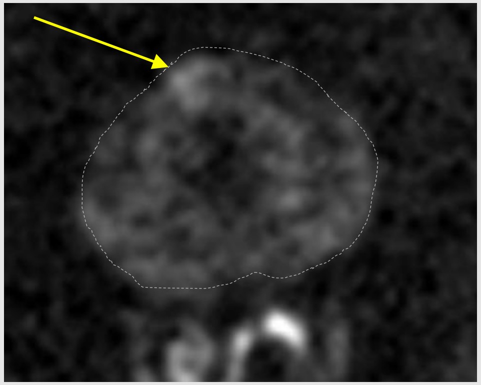	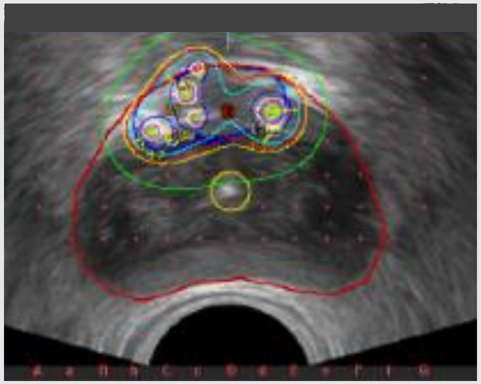	No re-recurrence

Transversal DWI at first recurrence with corresponding HDR-BT dose distribution.

No re-recurrence.

Site of re-recurrence: patient 7

Primary lesion | First recurrence | Dose distribution at Re - RT | Re-recurrence

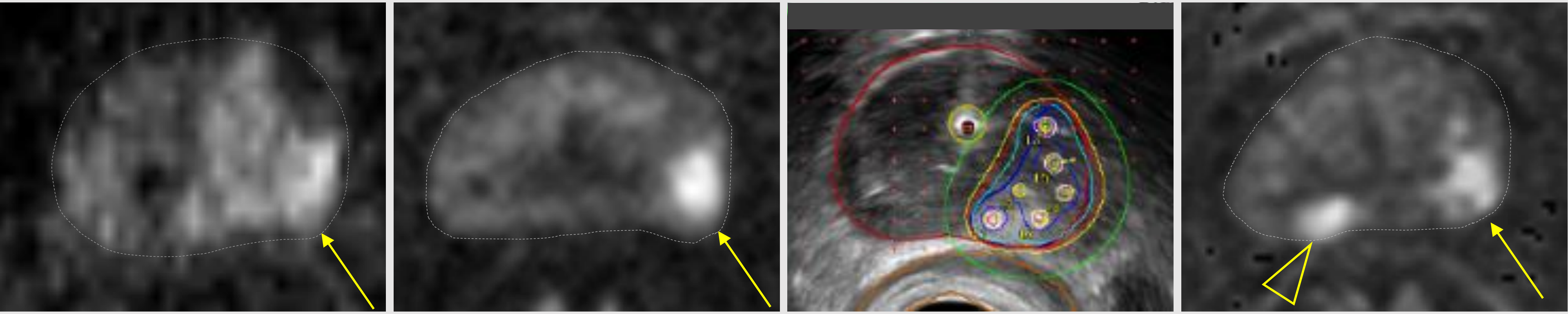

Transversal DWI of the primary lesion, first recurrence and re-recurrence with corresponding HDR-BT dose distribution.

Re-recurrence in the same site as the primary lesion and the first recurrence (arrows), and in a new site outside the re-irradiation high-dose region (arrowhead) near the margin of the primary lesion.

Site of re-recurrence: patient 8

Primary lesion

No prostate imaging

First recurrence

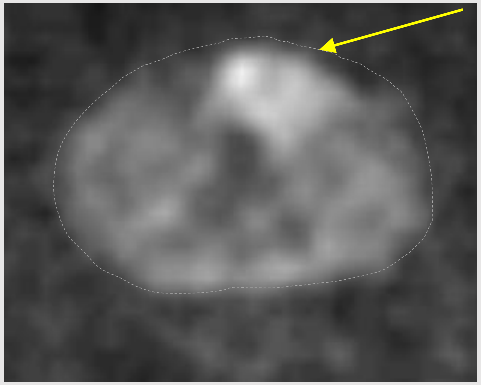

Dose distribution at Re - RT

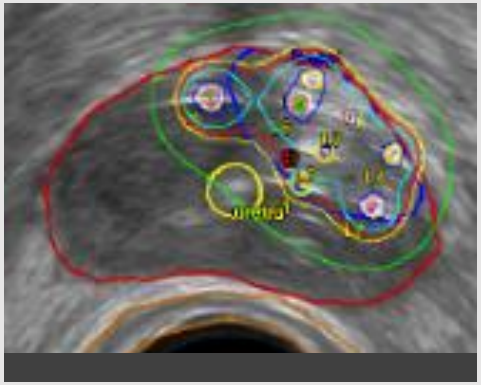

Re-recurrence

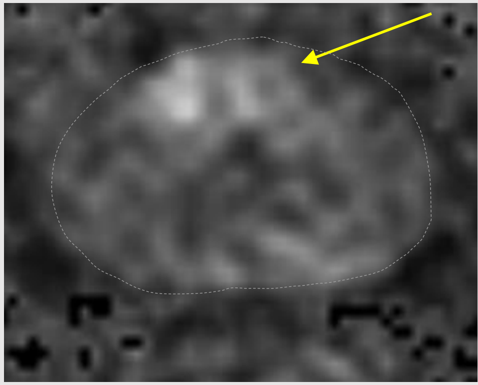

Transversal DWI of the first recurrence and re-recurrence with corresponding HDR-BT dose distribution.

Re-recurrence in the same site as the first recurrence.

Site of re-recurrence: patient 9

Primary lesion | First recurrence | Dose distribution at Re - RT | Re-recurrence

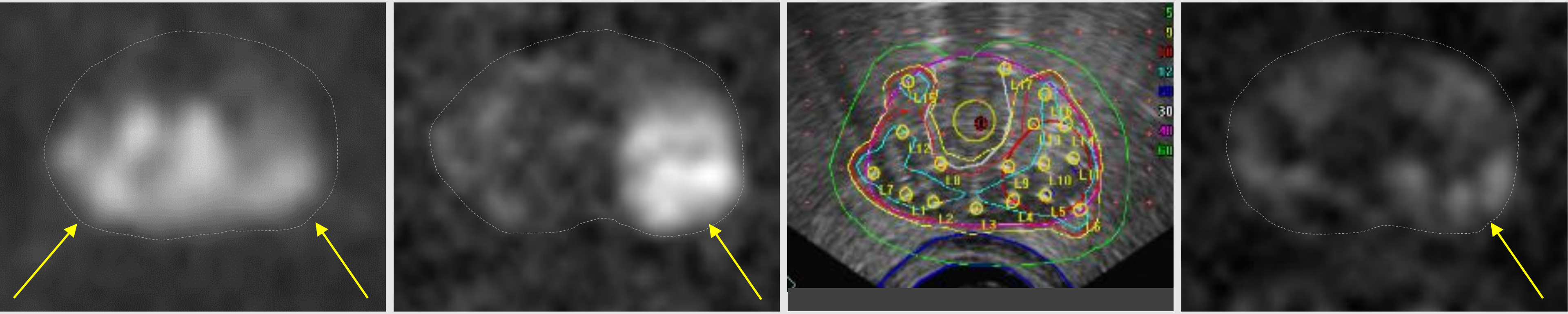

Transversal DWI of the primary lesion, first recurrence and re-recurrence with corresponding HDR-BT dose distribution.

Re-recurrence in the same site as the primary lesion and the first recurrence. Additional recurrence in the basis and seminal vesicles was not adequately covered by the re-irradiation high-dose region (not illustrated).

Site of re-recurrence: patient 10

Primary lesion	First recurrence	Dose distribution at Re - RT	Re-recurrence
No prostate imaging	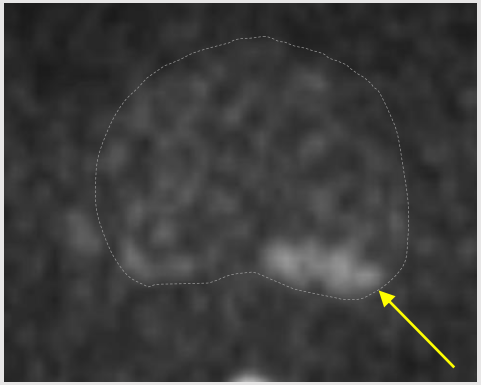	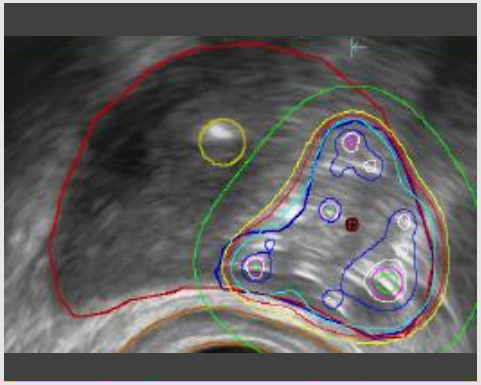	No prostate imaging

Transversal DWI of the first recurrence with corresponding HDR-BT dose distribution.

Site of re-recurrence could not be assessed.

Site of re-recurrence: patient 11

Primary lesion | First recurrence | Dose distribution at Re - RT | Re-recurrence

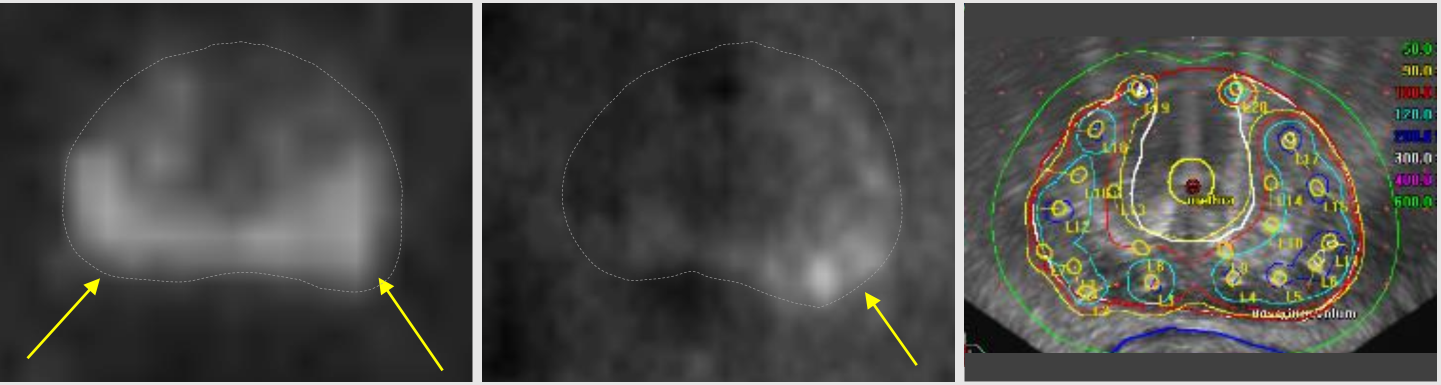

Inadequate imaging

Transversal DWI of the primary lesion and first recurrence with corresponding HDR-BT dose distribution.
DWI was inadequate at the right side at first recurrence and at both sides at re-recurrence due to a hip prosthesis.

The site of re-recurrence could not be assessed due to distortions from right hip prosthesis.

Site of re-recurrence: patient 12

Primary lesion | First recurrence | Dose distribution at Re - RT | Re-recurrence

No prostate imaging

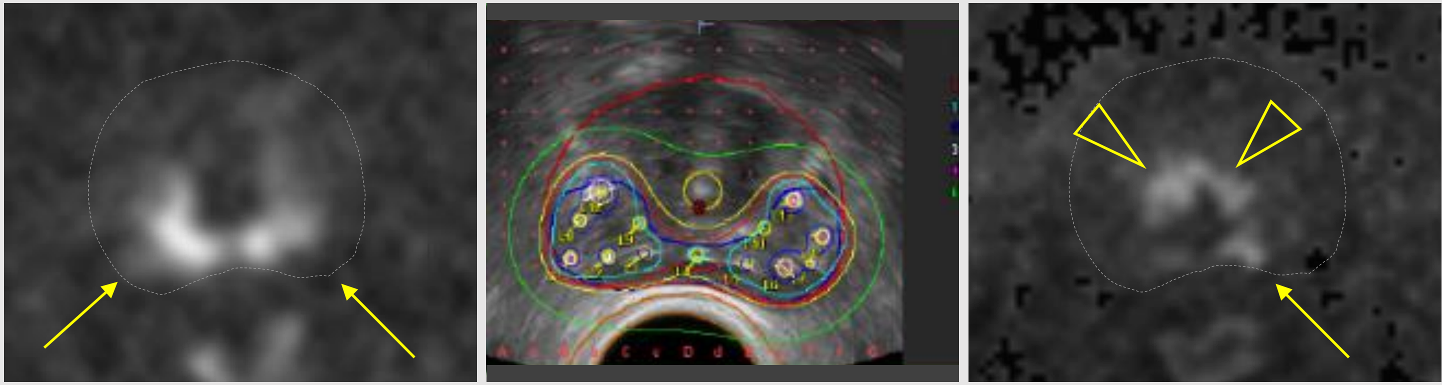

Transversal DWI of the first recurrence and re-recurrence with corresponding HDR-BT dose distribution.

Re-recurrence in the same site as the first recurrence (arrows).
Additional re-recurrence (arrowhead) in an area of peri-urethral dose sparing.

Site of re-recurrence: patient 13

Primary lesion

No prostate imaging

First recurrence

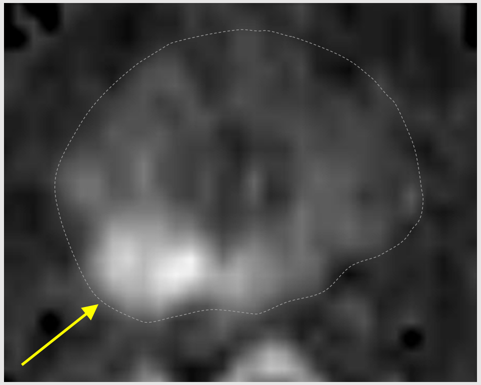

Dose distribution at Re - RT

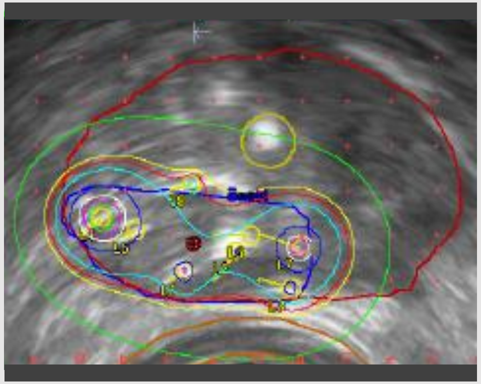

Re-recurrence

No prostate imaging

Transversal DWI of the first recurrence with corresponding HDR-BT dose distribution.

Site of re-recurrence could not be assessed.

Site of re-recurrences: patient 14

Primary lesion

No prostate imaging

First recurrence

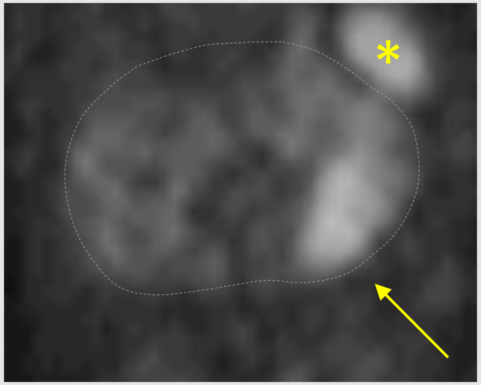

Dose distribution at Re - RT

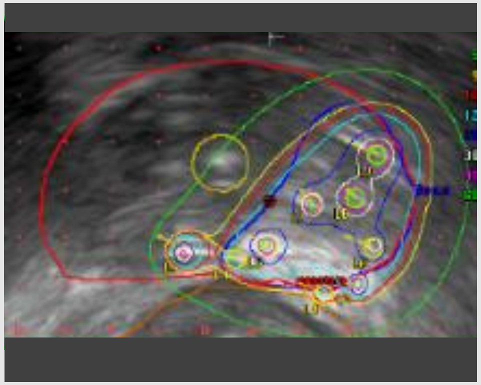

Re-recurrences

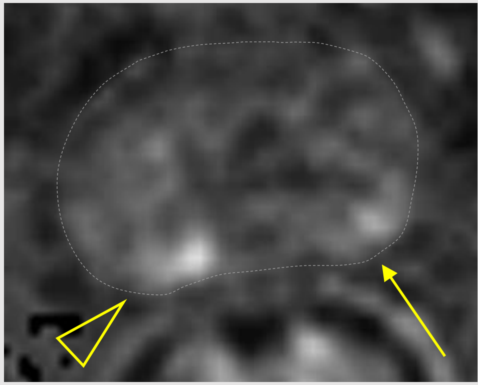

Transversal DWI of the first recurrence and re-recurrences with corresponding HDR-BT dose distribution.
A periprostatic vein with slow flow at first recurrence is marked with an asterix.

Re-recurrence in the same site as the first recurrence (arrows), and in a new site outside the re-irradiation high-dose region (arrowhead).

Site of re-recurrences: patient 15

Primary lesion | First recurrence | Dose distribution at Re - RT | Re-recurrences

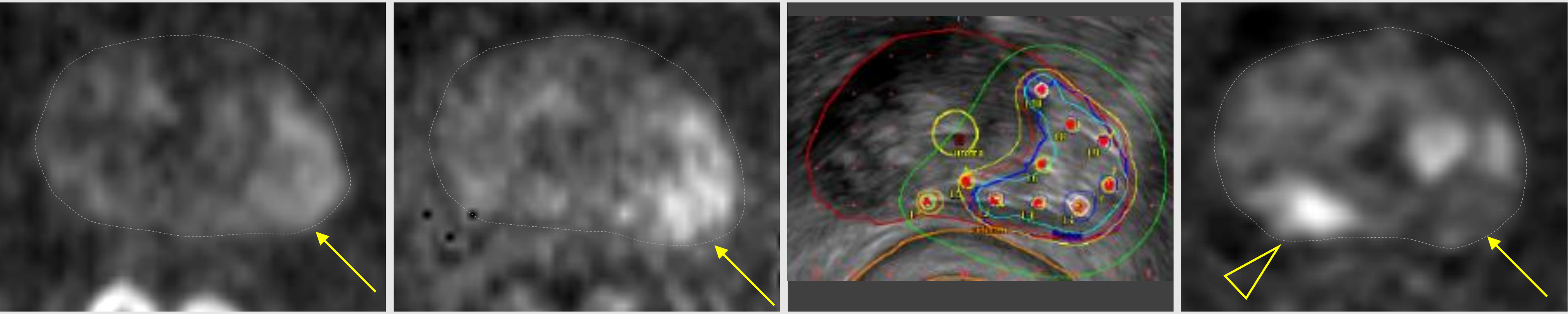

Transversal DWI of the primary lesion, first recurrence and re-recurrences with corresponding HDR-BT dose distribution.

Re-recurrence in the same site as the primary lesion and first recurrence (arrows), and in a new site outside the re-irradiation high-dose region (arrowhead).

Site of re-recurrence: patient 16

Primary lesion

No prostate imaging

First recurrence

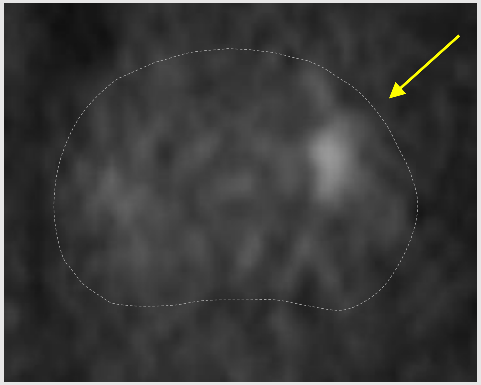

Dose distribution at Re - RT

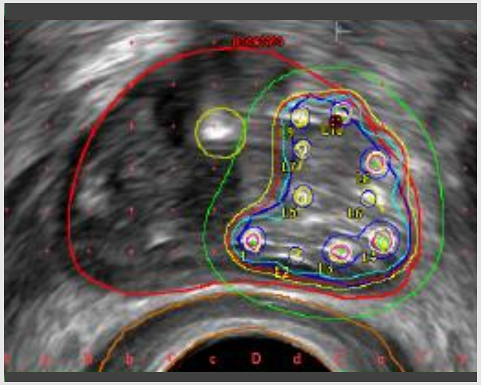

Re-recurrence

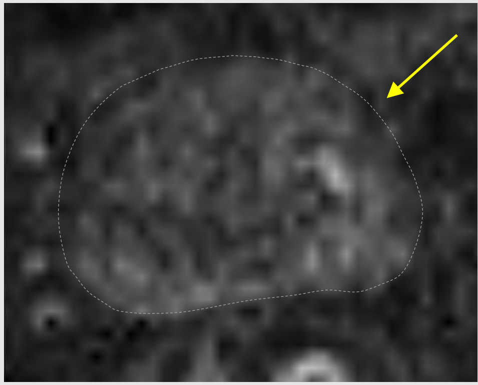

Transversal DWI of the first recurrence and re-recurrence with corresponding HDR-BT dose distribution.

Re-recurrence in the same site as the first recurrence.

Site of re-recurrence: patient 17

Primary lesion

No prostate imaging

First recurrence

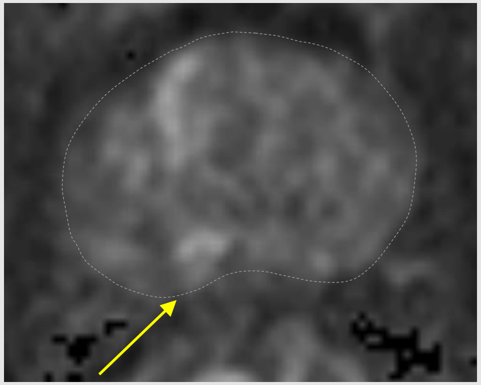

Dose distribution at Re - RT

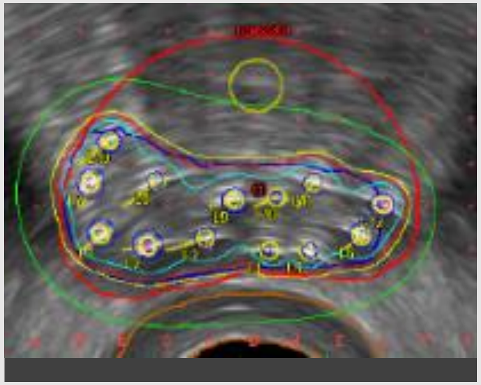

Re-recurrence

No prostate imaging

Transversal DWI of the first recurrence with corresponding HDR-BT dose distribution.

Site of re-recurrence could not be assessed.

Site of re-recurrence: patient 18

Primary lesion | First recurrence | Dose distribution at Re - RT | Re-recurrence

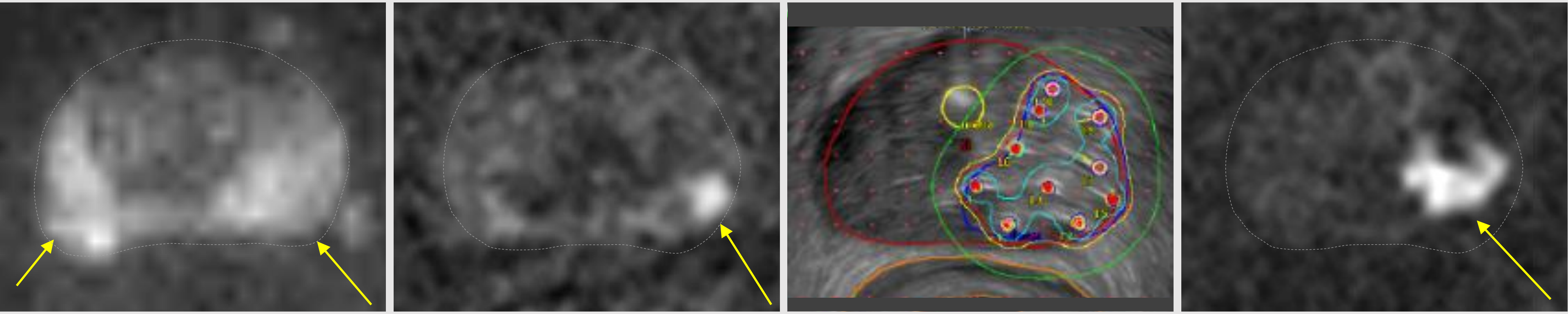

Transversal DWI of the primary lesion, first recurrence and re-recurrence with corresponding HDR-BT dose distribution.

Re-recurrence in the same site as the primary lesion and the first recurrence.

Site of re-recurrence: patient 19

Primary lesion | First recurrence | Dose distribution at Re - RT | Re-recurrence

No prostate imaging

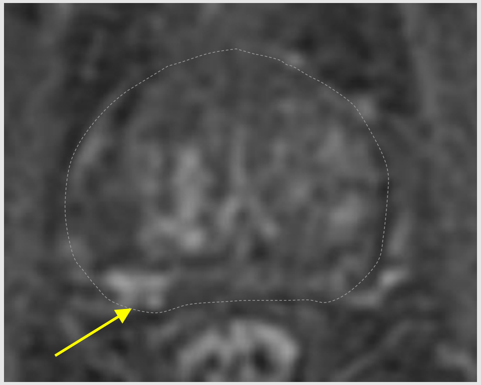

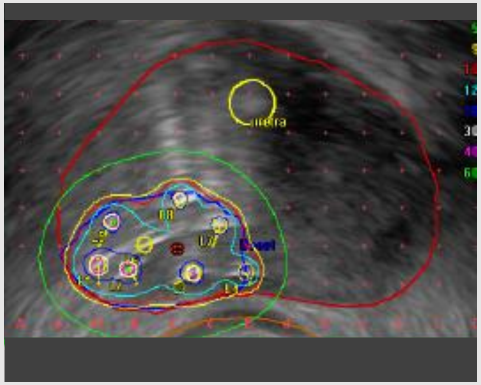

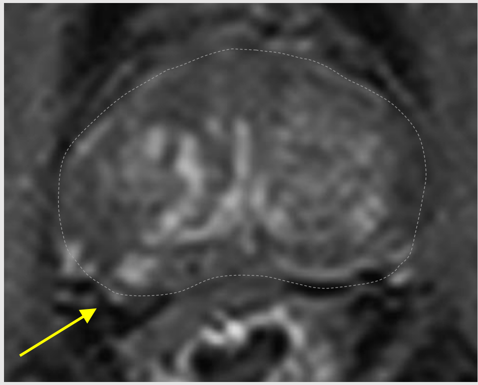

Transversal dynamic contrast-enhanced (DCE) sequences of the first recurrence and re-recurrence with corresponding HDR-BT dose distribution. The recurrence was sparsely visible at DWI, and is illustrated with DCE sequences.

Re-recurrence in the same site as the first recurrence.

Site of re-recurrence: patient 20

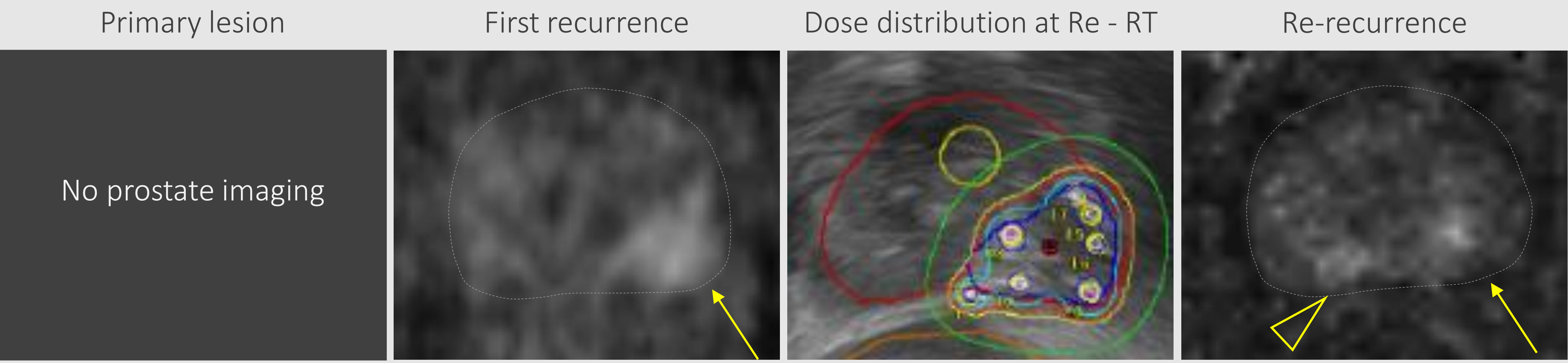

Transversal DWI of the first recurrence and re-recurrence with corresponding HDR-BT dose distribution.

Re-recurrence in the same site as the first recurrence (arrows), and in a new site outside the re-irradiation high-dose region (arrowhead).

Site of re-recurrence: patient 21

Primary lesion

No prostate imaging

First recurrence

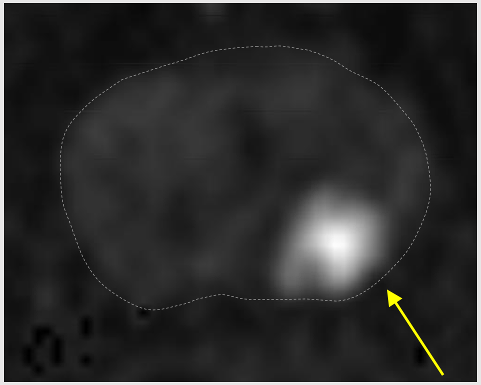

Dose distribution at Re - RT

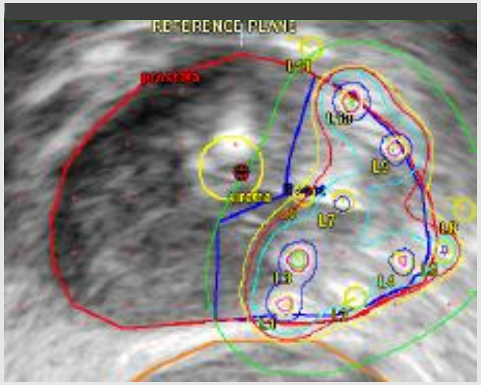

Re-recurrence

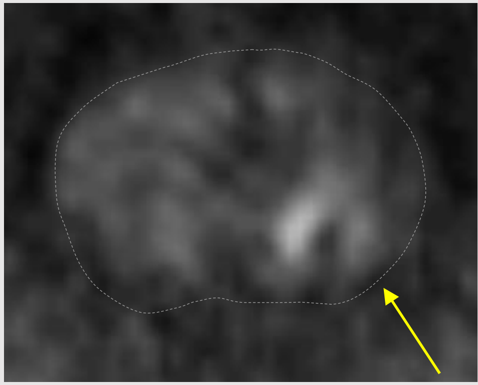

Transversal DWI of the first recurrence and re-recurrence with corresponding HDR-BT dose distribution.

Re-recurrence in the same site as the first recurrence.
Additional re-recurrence in the right lobe in the apical third, outside the re-irradiation high-dose region (not illustrated).

Site of re-recurrence: patient 22

Primary lesion	First recurrence	Dose distribution at Re - RT	Re-recurrence
No prostate imaging	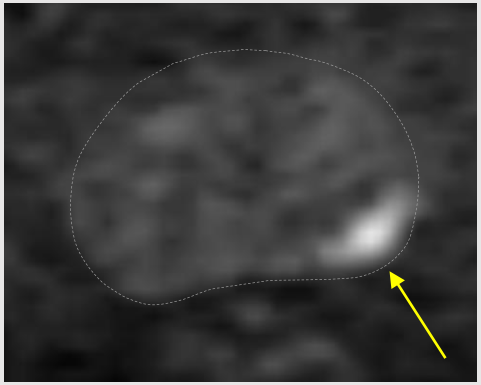	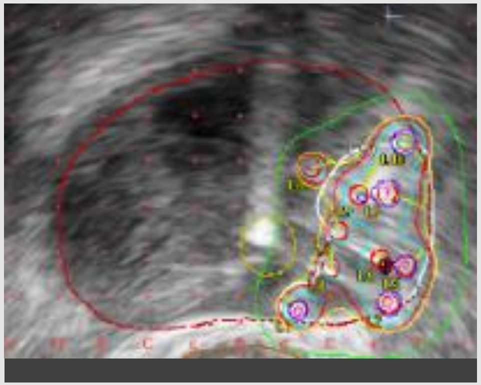	No re-recurrence

Transversal DWI of the first recurrence with corresponding HDR-BT dose distribution.

No re-recurrence.

Site of re-recurrence: patient 23

Primary lesion | First recurrence | Dose distribution at Re - RT | Re-recurrence

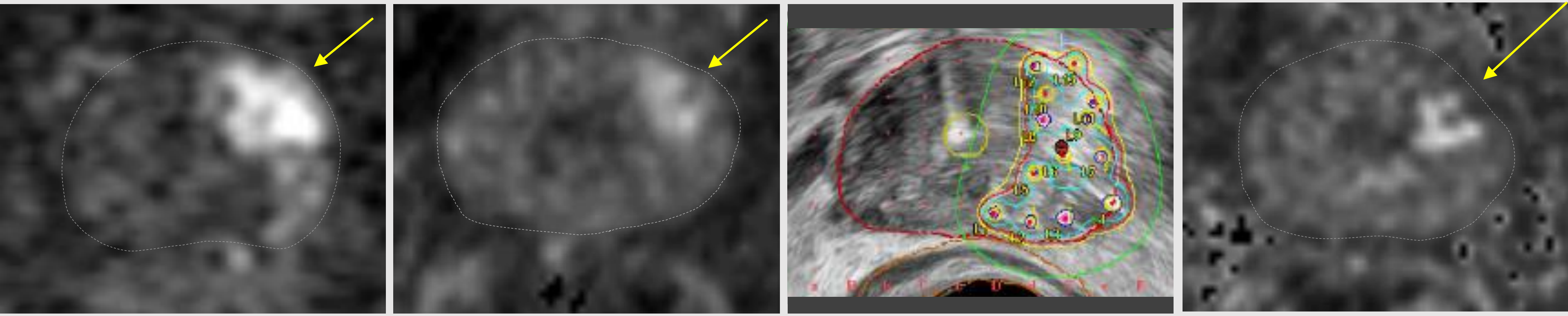

Transversal DWI of the primary lesion, first recurrence and re-recurrence with corresponding HDR-BT dose distribution.

Re-recurrence in the same site as the primary lesion and the first recurrence. Additional re-recurrence in the seminal vesicles outside the re-irradiation high-dose region (not illustrated).

Site of re-recurrence: patient 24

Primary lesion | First recurrence | Dose distribution at Re - RT | Re-recurrence

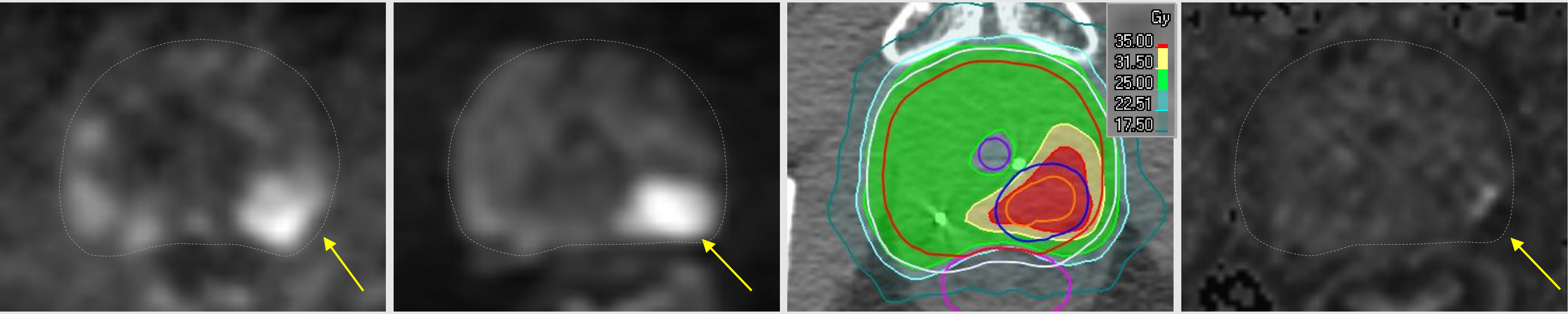

Transversal DWI of the primary lesion, first recurrence and re-recurrence with corresponding SBRT plan.

Re-recurrence in the same site as the primary lesion and the first recurrence. Additional re-recurrence in the left seminal vesicle outside the re-irradiation high-dose region (not illustrated).

Site of re-recurrence: patient 25

Primary lesion	First recurrence	Dose distribution at Re - RT	Re-recurrence
No prostate imaging	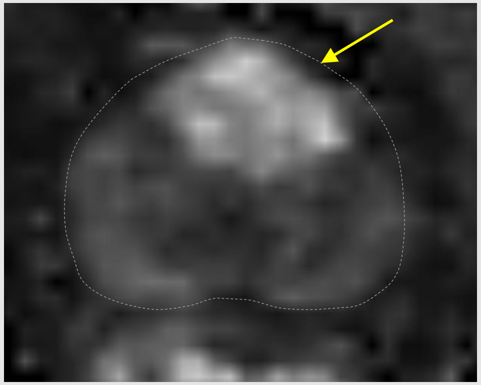	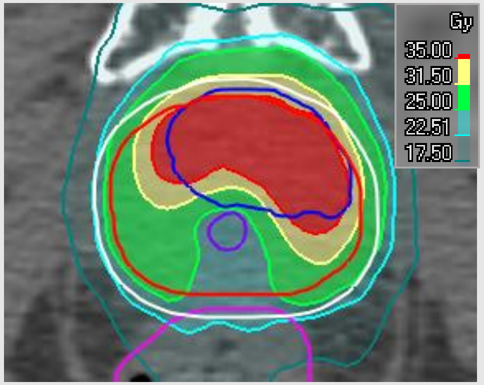	No prostate imaging

Transversal DWI of the re-recurrence with corresponding SBRT plan.

Site of re-recurrence could not be assessed.

Site of re-recurrence: patient 26

Primary lesion | First recurrence | Dose distribution at Re - RT | Re-recurrence

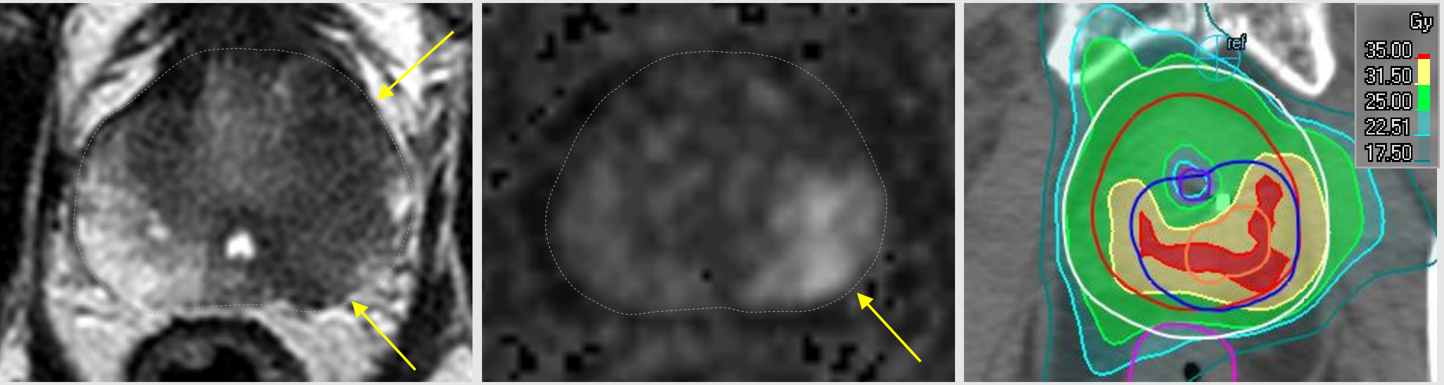

No re-recurrence

Transversal T2W of the primary lesion and DWI of the first recurrence with corresponding SBRT plan.

No re-recurrence.

Site of re-recurrence: patient 27

Primary lesion	First recurrence	Dose distribution at Re - RT	Re-recurrence
No prostate imaging	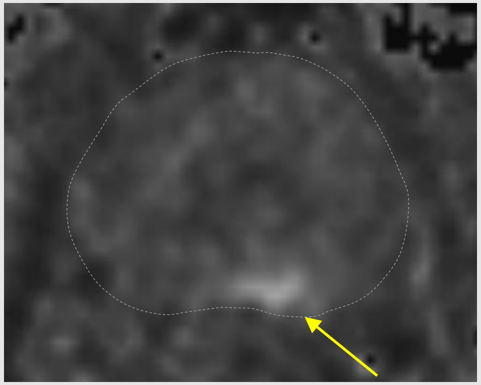	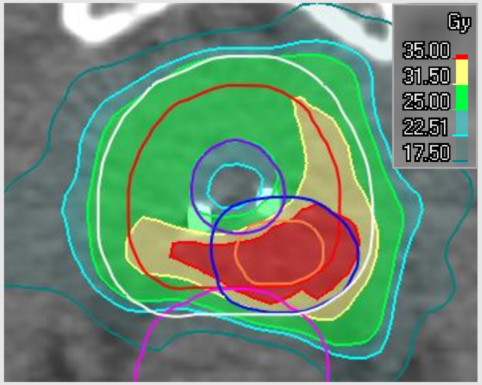	No re-recurrence

Transversal DWI of the first recurrence with corresponding SBRT plan.

No re-recurrence.

Site of re-recurrence: patient 28

Primary lesion | First recurrence | Dose distribution at Re - RT | Re-recurrence

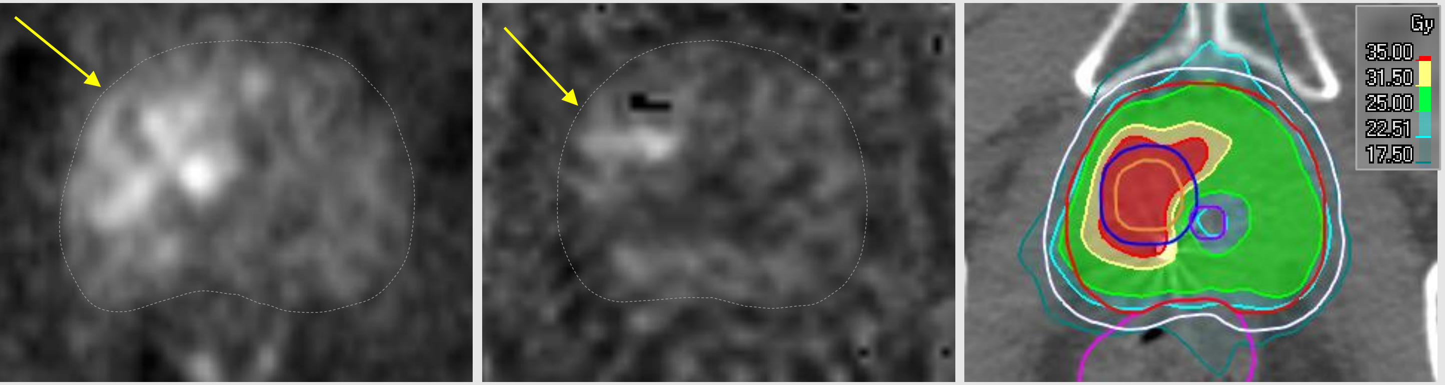

No re-recurrence

Transversal DWI of the primary lesion and first recurrence with corresponding SBRT plan.

No re-recurrence.

Site of re-recurrence: patient 29

Primary lesion | First recurrence | Dose distribution at Re - RT | Re-recurrence

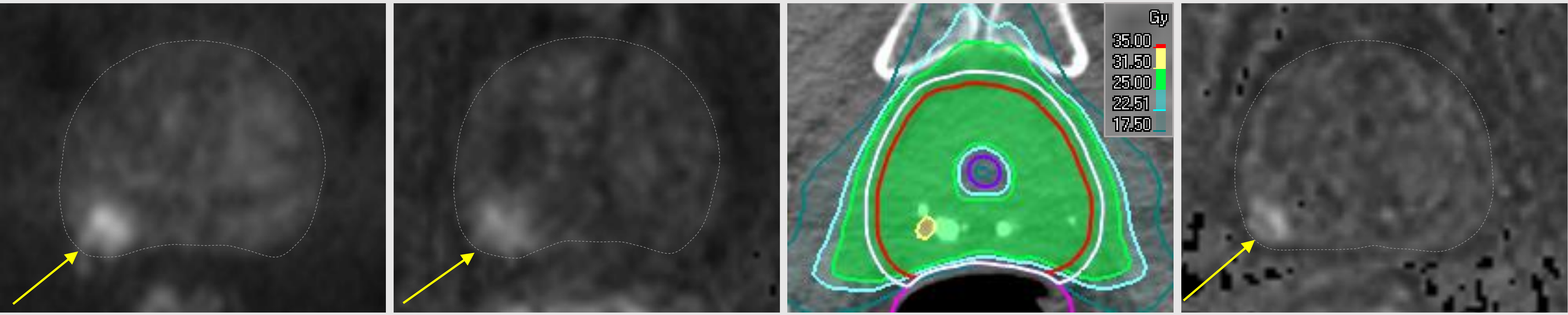

Transversal DWI of the primary lesion, first recurrence and re-recurrence with corresponding SBRT plan.

Re-recurrence in the same site as the primary lesion and the first recurrence.

Site of re-recurrence: patient 30

Primary lesion | First recurrence | Dose distribution at Re - RT | Re-recurrence

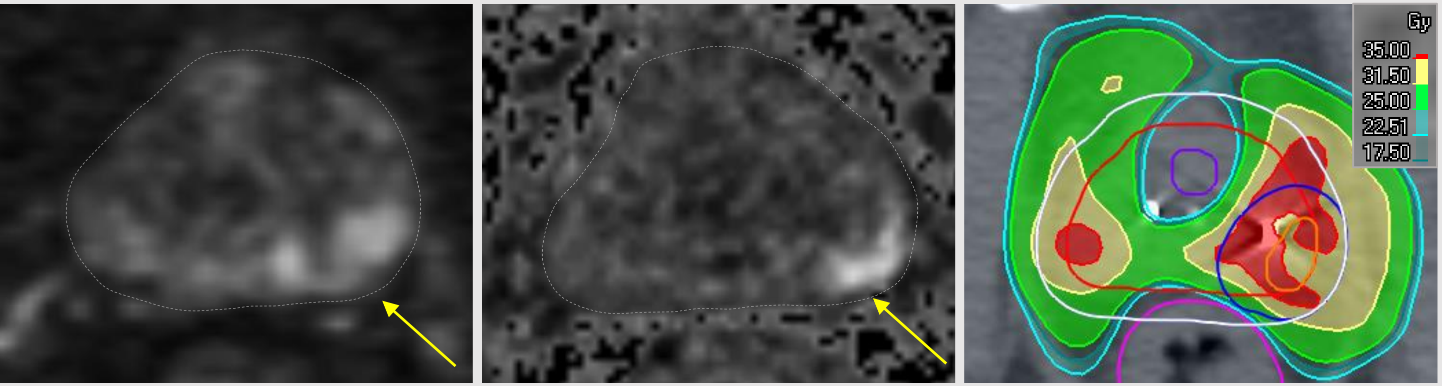

No re-recurrence

Transversal DWI of the primary lesion and first recurrence with corresponding SBRT plan.

No re-recurrence.

Site of re-recurrence: patient 31

Primary lesion | First recurrence | Dose distribution at Re - RT | Re-recurrence

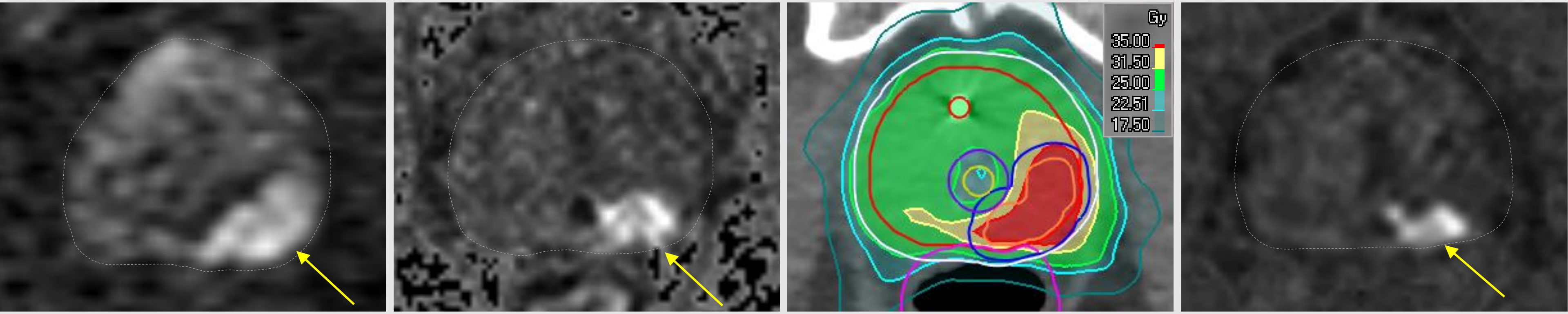

Transversal DWI of the primary lesion, first recurrence and re-recurrence with corresponding SBRT plan.

Re-recurrence in the same site as the primary lesion and the first recurrence. Additional re-recurrence in the seminal vesicles outside the re-irradiation high-dose region (images not shown).

Site of re-recurrence: patient 32

Primary lesion | First recurrence | Dose distribution at Re - RT | Re-recurrence

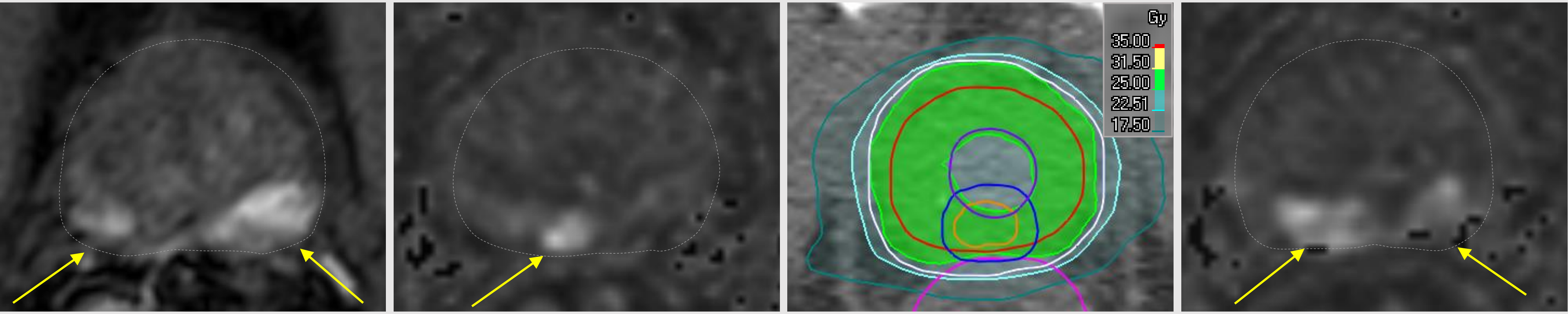

Transversal DCE of the primary lesion, DWI of the first recurrence and re-recurrence with corresponding SBRT plan.

Re-recurrence in the same site as the primary lesion and the first recurrence.

Site of re-recurrence: patient 33

Primary lesion | First recurrence | Dose distribution at Re - RT | Re-recurrence

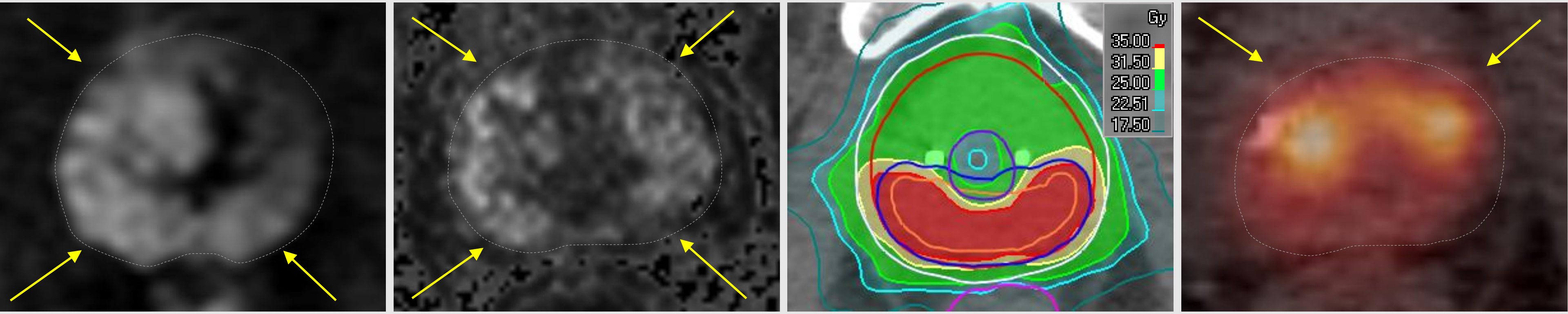

Transversal DWI of the primary lesion and first recurrence, prostate-specific membrane antigen (PSMA) PET/CT of the re-recurrence with corresponding stereotactic dose distribution plan.

Re-recurrence in the same site as the primary lesion and first recurrence (arrows), outside the re-irradiation high-dose region.
