# Supplementary material for: Image-Based Recurrence Patterns After Reirradiation in Prostate Cancer with Long-Term Follow-Up
Source: Adv Radiat Oncol. 2025 Sep 16;10(12):101900. doi: 10.1016/j.adro.2025.101900 (PMC12596965; doi:10.1016/j.adro.2025.101900)
Supplement: Survival plots_anon [file mmc2.docx]

# Survival plots

|  |
| --- |
| Overall survival – all patients |
|  |
| Prostate cancer specific survival - all patients. |

|  |
| --- |
|  |
|  |
| Biochemical Recurrence Free Survival by D’Amico risk group |
|  |
|  |
|  |
|  |
